# Prognostic Modeling of 1-Year Survival in Hepatocellular Carcinoma: A Comparative Evaluation of Machine Learning Methods with Class Balancing

**DOI:** 10.3390/diseases14100377

**Published:** 2026-10-13

**Authors:** Jinlian Wang, Hui Li, Changqing Ju, Hongfang Liu

**Affiliations:** 1 Department of Quantitative and Systems Health Sciences, University of Texas at Austin Dell Medical School, Austin, TX 78712, USA; jinlian.wang@austin.utexas.edu; 2 Department of Anesthesiology, McGovern Medical School UTHealth, Houston, TX 77030, USA; hui.li@austin.utexas.edu (H.L.); changqing.ju@uth.tmc.edu (C.J.)

**Keywords:** hepatocellular carcinoma, survival prediction, machine learning, SMOTE, SHAP, diabetes mellitus, chronic liver disease, EASL guidelines, model interpretability

## Abstract

Objective: To develop and comparatively evaluate machine learning models for predicting 1-year survival in hepatocellular carcinoma (HCC) patients, focusing on the prognostic interplay between chronic liver diseases and diabetes aligned with the EASL Clinical Practice Guidelines (2018), and to assess the impact of class balancing via Synthetic Minority Oversampling Technique (SMOTE) on model performance. Methods: Retrospective analysis was performed on the UCI HCC Survival Dataset (*N* = 165). Following median/mode imputation of ~10% missing values, four classifiers were evaluated: Logistic Regression (LR), Multilayer Perceptron (MLP), Gradient Boosting (GB), and Random Forest (RF), each trained with and without SMOTE oversampling (eight variants total). Models were trained on 16 EASL-aligned clinical and biochemical features using a stratified 80/20 train/test split (training *N* = 132, held-out test *N* = 33). Performance was evaluated via accuracy, AUC-ROC (with 95% bootstrap confidence intervals across 1000 iterations), precision, recall, and macro F1-score. Stratified 5-fold cross-validation and multiple imputation sensitivity analyses (MissForest, KNN, MICE, complete-case) were conducted. SHAP (SHapley Additive exPlanations) was applied to the top-performing model to provide patient-level interpretability. Results: Diabetes was present in 33.9% of patients (56/165; 28/102 survivors [27.5%] vs. 28/63 non-survivors [44.4%]). Diabetes was significantly associated with 1-year mortality in univariate analysis (χ^2^ = 5.02, *p* = 0.025) but not after multivariable adjustment (aOR = 0.50, 95% CI: 0.21–1.22, *p* = 0.129). GB + SMOTE achieved the highest discriminative ability (AUC-ROC: 0.727, 95% CI: 0.552–0.886), while LR + SMOTE yielded the highest overall accuracy (72.7%) and macro F1-score (0.723). Without SMOTE, all models exhibited poor minority-class detection (non-survival F1 range: 0.300–0.615). SMOTE markedly improved non-survival recall across LR (0.231 to 0.769) and GB (61.5% to 69.2%). Stratified 5-fold CV AUC on the training set was highest for RF (0.779 ± 0.100), followed by GB (0.744 ± 0.065). TreeSHAP analysis identified ECOG performance status (mean |SHAP| = 1.284) and ALT (1.060) as the most influential predictors; diabetes ranked 15th (mean |SHAP| = 0.014), consistent with minimal independent predictive contribution. Conclusions: Gradient boosting and regularized linear regression combined with SMOTE class balancing provide competitive, well-calibrated, and transparent risk stratification on small, imbalanced tabular HCC datasets, offering an exploratory proof-of-concept for personalized prognostic modeling that requires external validation.

## Simple Summary

Accurate prediction of 1-year survival is critical for informing therapeutic decision-making in patients with hepatocellular carcinoma (HCC) arising on a background of chronic liver disease. In this retrospective study of 165 HCC patients from the UCI repository, we comparatively evaluated eight machine learning workflows combining logistic regression, neural networks, and tree-based ensembles with Synthetic Minority Oversampling (SMOTE). We found that gradient boosting combined with SMOTE achieved the highest discriminative ability (AUC-ROC: 0.727, 95% CI: 0.552–0.886; Brier score: 0.182), and logistic regression with SMOTE yielded 72.7% accuracy, substantially improving the detection of high-risk non-surviving patients (recall: 76.9%). Deep learning (MLP) did not outperform ensemble methods on this tabular dataset. SHAP interpretability identified performance status and liver enzymes as dominant predictors, while diabetes contributed negligibly as an independent feature. This study provides an interpretable, guideline-aligned machine learning framework for clinical risk stratification in HCC.

## 1. Introduction

Hepatocellular carcinoma (HCC) is the fifth most common malignancy and the second leading cause of cancer-related mortality worldwide, accounting for approximately 90% of primary liver cancers [1]. Its incidence continues to rise, particularly in populations with a high burden of chronic liver diseases including cirrhosis, chronic viral hepatitis (HBV/HCV), and metabolic dysfunction-associated steatohepatitis (MASH) [2,3,4,5,6]. In these settings, type 2 diabetes mellitus acts as a frequent metabolic comorbidity that accelerates hepatic fibrogenesis and carcinogenesis via insulin resistance, chronic hyperinsulinemia, and systemic low-grade inflammation [2]. Despite therapeutic advances in surgical resection, liver transplantation, and locoregional therapies, the 5-year overall survival rate for HCC remains below 30%, largely due to late clinical presentation and underlying hepatic decompensation [1,7,8].

The European Association for the Study of the Liver (EASL) and American Association for the Study of Liver Diseases (AASLD) Clinical Practice Guidelines emphasize the compounding prognostic impact of metabolic risk factors in chronic liver disease, recommending risk-stratified management [1]. Conventional staging systems, including the Barcelona Clinic Liver Cancer (BCLC) classification and the Child–Pugh and ALBI scoring systems, combine tumor burden, synthetic liver reserve, and performance status to guide clinical decision-making, achieving moderate prognostic accuracy (AUC: 0.70–0.80) [9,10,11,12]. However, these linear scoring systems cannot capture subtle non-linear interactions among hepatic enzymes, systemic inflammation, and metabolic comorbidities [11,13,14].

Computational machine learning methods offer significant potential for refining prognostic stratification in oncology [15,16,17,18,19]. Santos et al. [9] previously demonstrated the utility of cluster-based oversampling and logistic regression on the UCI HCC Survival Dataset (AUC: 0.70). Subsequent investigations have applied random forests and support vector machines for prognostic prediction in viral hepatitis cohorts, yielding AUCs of 0.80–0.90 [3,20,21,22]. Furthermore, deep learning neural networks have shown substantial utility in radiomics and digital pathology [4,22,23]. However, recent theoretical benchmarks demonstrate that for small-to-moderate tabular clinical datasets characterized by feature heterogeneity and missingness, tree-based ensembles frequently match or outperform deep neural architectures [24,25].

A critical methodological barrier in HCC prognostic modeling is class imbalance, where 1-year survivors outnumber deceased patients, biasing classifiers toward the majority class and leading to high false-negative rates for high-risk patients [26]. While the Synthetic Minority Oversampling Technique (SMOTE) [26] addresses this limitation in general clinical domains [27,28], systematic comparisons of how SMOTE influences diverse model paradigms spanning linear classifiers, multi-layer neural networks, and tree ensembles remain sparse.

We address three questions: (1) Which machine learning approach best predicts 1-year survival in diagnosed HCC patients on a small guideline-aligned tabular dataset? (2) What is the impact of SMOTE class balancing across different model architectures? (3) Which clinical features are most predictive, and what do SHAP-based explanations reveal about diabetes and chronic liver disease interactions at the individual patient level? Our contribution is fourfold: (1) systematic comparative evaluation of eight model variants on EASL-aligned features; (2) evaluation of how SMOTE class balancing affects minority-class (non-survivor) detection across model families; (3) an assessment showing that the MLP did not outperform tree-based or linear models on this small tabular dataset; and (4) patient-level SHAP interpretability of the best model, providing transparent, patient-level feature explanation.

## 2. Materials and Methods

### 2.1. Cohort and Clinical Dataset

Data were obtained from the publicly accessible UCI HCC Survival Dataset [9], comprising retrospective clinical records of 165 patients diagnosed with HCC at a university hospital in Portugal between 2008 and 2014. The dataset contains 49 clinical, biochemical, and pathological features selected per EASL-EORTC guidelines [1], comprising 23 quantitative variables (e.g., alpha-fetoprotein [AFP], transaminases, bilirubin, albumin) and 26 qualitative variables (e.g., viral hepatitis status, diabetes, cirrhosis, ascites). The primary clinical endpoint was binary 1-year overall survival (1 = alive at 1 year, 0 = deceased within 1 year). The cohort exhibits moderate class imbalance, with 102 survivors (61.8%) and 63 non-survivors (38.2%). Missing values constituted 10.22% across all entries.

### 2.2. Data Preprocessing, Missingness Diagnoses and Feature Selection

Data preprocessing was conducted in Python (version 3.13.13) using Pandas (version 4.0.6) and Scikit-learn (version 1.9.1) [29] and PyTorch (version 1.14.1) [30]. Little’s Missing Completely at Random (MCAR) test across quantitative baseline covariates did not reject the MCAR hypothesis (*p* = 0.082) [31]. Because this test has limited power at *N* = 165, we assumed a missing-at-random (MAR) mechanism and assessed robustness with alternative imputation strategies. In the primary predictive pipeline, missing values were imputed using feature medians for continuous variables and modes for categorical variables, with imputation parameters estimated within training partitions only. For the descriptive and multivariable analyses (Table 1 and Table 2), the same imputation was applied to the full cohort. Sensitivity analyses were conducted comparing median/mode imputation against k-Nearest Neighbors (k-NN, *k* = 5), Multiple Imputation by Chained Equations (MICE, *m* = 5) [31], MissForest [32], and complete-case analysis (*N* = 118). Sixteen biologically salient variables were selected based on EASL guidelines: Perf. Status (ECOG), ALT, Platelets, AST, Ascites, HBeAg, Hemoglobin, Albumin, HBcAb, HCVAb, AFP, NASH, Cirrhosis, HBsAg, Diabetes Mellitus, and Alcohol Abuse. Continuous variables were scaled using RobustScaler [29] to center features by their median and scale them by the interquartile range (IQR).

### 2.3. Model Architectures and Hyperparameter Selection

To predict 1-year HCC survival, we evaluated four complementary machine learning model families spanning linear models, deep neural networks, and tree-based ensembles:

**Multivariable and Regularized Logistic Regression (LR):** (a) Explanatory model. An unpenalized multivariable maximum-likelihood model (statsmodels Logit [19]) was fitted on the full cohort (*N* = 165) after median/mode imputation to estimate adjusted odds ratios (aORs) for 15 of the 16 EASL features (Table 2). HBeAg was excluded because only one patient was positive (complete separation), and AST, ALT, and AFP were natural-log transformed. (b) Predictive model. A regularized logistic regression classifier (*L2* penalty, *C* = 1.0; scikit-learn [29]) was trained within the 80/20 train/test framework on all 16 RobustScaler-transformed features and served as the linear baseline.

**Multilayer Perceptron (MLP):** A feedforward MLP was implemented in PyTorch [30]. Its architecture was an input layer (*d* = 16 features); hidden layer 1 with 32 units, batch normalization, ReLU activation, and dropout (*p* = 0.20); hidden layer 2 with 16 units and ReLU activation; and a single sigmoid output giving the predicted probability of 1-year survival (Figure 1). The network was trained with the Adam optimizer (learning rate = 0.005, *L2* weight decay = 1 × 10^−4^) to minimize binary cross-entropy, using a batch size of 32 for up to 50 epochs. Early stopping (patience = 10 epochs) monitored the loss on a validation subset.

**Gradient Boosting (GB):** Gradient boosting (GradientBoostingClassifier [29]), based on Friedman’s gradient boosting framework [33], builds an additive ensemble of shallow decision trees, each fitted to the pseudo-residuals of the binary cross-entropy loss. Tree-based splits capture non-linear thresholds and biomarker interactions without manually specified interaction terms. The final configuration was 100 estimators, maximum depth = 3, learning rate = 0.10, and subsample = 0.80.

**Random Forest (RF):** Random Forest (RandomForestClassifier [29]), based on Breiman’s randomized bagging ensemble [34], aggregates decorrelated decision trees grown on bootstrap samples, with randomly selected features considered at each split. The final configuration was 200 trees, maximum depth = 5, and a minimum of four samples per split.

**Hyperparameter selection**. Given the small training cohort (*N* = 132), hyperparameters were fixed a priori to commonly used, conservative values for small tabular datasets (shallow trees, moderate ensemble sizes, dropout and weight decay for the MLP) rather than extensively tuned, to limit the risk of overfitting to the tuning folds. Model stability was assessed by stratified 5-fold cross-validation (Table 3).

**Synthetic Minority Oversampling Technique (SMOTE).** To address class imbalance (38.2% non-survivors vs. 61.8% survivors), SMOTE [26] was applied only to the training partition (*N* = 132 → 164 samples), synthesizing minority-class samples by interpolation between *k* = 5 nearest neighbors. The held-out test set (*N* = 33) was not resampled, to avoid optimistic bias from data leakage.

### 2.4. Evaluation Metrics

Model performance was comprehensively quantified on the held-out test set (*N* = 33, 13 deceased, 20 survivors) across eight clinical and statistical metrics: Accuracy, Area Under the Receiver Operating Characteristic Curve (AUC-ROC) with 95% bootstrap confidence intervals derived across 1000 resampling iterations, Precision (Positive Predictive Value), Recall/Sensitivity (True Positive Rate), Specificity (True Negative Rate), Non-Survival F1-Score, Macro-Averaged F1-Score, and Brier Score [35]. Additionally, stratified 5-fold cross-validation was conducted across 10 random seeds on the training set (*N* = 132) to benchmark out-of-fold generalization. Pairwise differences in test-set AUC between GB + SMOTE and each comparator model were assessed using DeLong’s nonparametric test for correlated ROC curves [12]. For the baseline comparisons in Table 1, continuous variables were compared between survivors and non-survivors using the two-sample Student’s *t*-test with pooled variance, and the accompanying correlation coefficient (r) is the point-biserial Pearson correlation with 1-year survival, which is derived from the same comparison and therefore has an identical *p*-value. Categorical variables were compared using Pearson’s χ^2^ test without continuity correction, or two-sided Fisher’s exact test when any expected cell count was <5 (NASH and HBeAg); for these variables, r is the phi coefficient. Two-sided *p* < 0.05 was considered statistically significant.

### 2.5. SHAP Interpretability Analysis

To evaluate feature contributions and provide patient-level transparency, we integrated SHapley Additive exPlanations (SHAP) grounded in cooperative game theory [36]. TreeSHAP was utilized on the top-performing tree ensemble (GB + SMOTE) to compute exact Shapley values on the log-odds scale, assessing both global feature importance (mean |SHAP|) and directional biomarker effects (e.g., evaluating the independent prognostic impact of diabetes relative to liver enzymes and performance status). TreeSHAP values were computed on the held-out test set (*N* = 33), using the GB + SMOTE model trained on the SMOTE-augmented training partition, so that explanations reflect patients unseen during training. Global importance was summarized as the mean absolute SHAP value across test-set patients.

## 3. Results

### 3.1. Baseline Cohort Demographics and Clinical Characteristics

Exploratory data analysis was conducted to characterize the cohort and the distribution of key risk factors related to chronic liver disease and diabetes. The cohort (*N* = 165) had a mean age of 67.0 ± 11.2 years and a male predominance (70.3%), consistent with the demographic profile of HCC in Western populations. Cirrhosis was present in 90.3% of patients, in keeping with its role as the principal precursor of HCC [1]. Diabetes mellitus was present in 33.9% of patients (56/165; 28/102 survivors [27.5%] vs. 28/63 non-survivors [44.4%]) and was associated with 1-year mortality in univariate analysis (χ^2^ = 5.02, *p* = 0.025; *r* = −0.174). Higher serum albumin (3.54 ± 0.61 vs. 3.07 ± 0.62 g/dL, *p* < 0.0001) and platelet count (178.5 ± 92.4 vs. 141.0 ± 78.3 × 10^9^/L, *p* = 0.008) were associated with survival, whereas ECOG PS ≥ 1 (19.6% vs. 65.1%, *p* < 0.0001) and ascites (10.8% vs. 38.1%, *p* < 0.0001) were associated with mortality (Table 1, Figure 2).

### 3.2. Correlation Structure and Clinical Marker Associations

Pairwise Pearson correlations among the EASL clinical features and 1-year survival are shown in Figure 3. Inter-feature correlations were weak to moderate (|*r*| ≤ 0.66), the strongest being between alcohol abuse and AFP (*r* = 0.66) and between performance status and ascites (*r* = 0.47), indicating limited multicollinearity among predictors. The correlation matrix was computed on the imputed feature matrix used for model development; univariate associations with 1-year survival, including their direction and statistical significance, are reported in Table 1.

### 3.3. Multivariable Logistic Regression Analysis

In the multivariable model including 15 EASL features (Table 2), higher hemoglobin was independently associated with 1-year survival (aOR = 1.33 per g/dL, 95% CI: 1.06–1.67, *p* = 0.014), whereas higher log-AST (aOR = 0.23, 95% CI: 0.08–0.66, *p* = 0.006) and log-AFP (aOR = 0.79, 95% CI: 0.69–0.91, *p* = 0.001) were independently associated with lower odds of survival. ECOG PS ≥ 1 (aOR = 0.40, *p* = 0.070) and log-ALT (aOR = 2.51, *p* = 0.064) showed borderline associations. Albumin, ascites, and platelet count, which differed significantly between groups in univariate analysis (Table 1), were not independently associated with survival after adjustment. Diabetes did not reach statistical significance (aOR = 0.50, 95% CI: 0.21–1.22, *p* = 0.129).

### 3.4. Subgroup Analysis of AST and Diabetes Interplay

Figure 4 shows serum AST levels stratified by 1-year survival and diabetes status. Median AST values were similar across the four subgroups (non-diabetic survivors, 94.5 U/L; diabetic survivors, 96.0 U/L; non-diabetic deceased, 95.2 U/L; diabetic deceased, 95.8 U/L), and no clear interaction between diabetes and AST was apparent at this sample size.

### 3.5. Deep Learning Training Dynamics

Training and validation convergence curves for the Multilayer Perceptron (MLP) over 50 epochs demonstrate that the binary cross-entropy loss smoothly declined from 0.69 to 0.42 on the training set, while validation loss plateaued at ~0.51 after 35 epochs, at which point early stopping was triggered to limit overfitting.

### 3.6. Comparative Classifier Performance and Impact of SMOTE

Table 4 details held-out test performance (*N* = 33). GB + SMOTE achieved highest discriminative AUC (0.727, 95% CI: 0.552-0.886; Brier: 0.182), while LR + SMOTE yielded 72.7% accuracy and macro F1 of 0.723. Pairwise DeLong tests confirmed no significant difference in AUC between GB + SMOTE and LR + SMOTE (*p* = 0.779), RF (*p* = 0.660), or baseline GB (*p* = 0.562). In standard un-augmented models, severe class imbalance caused high false-negative rates: baseline LR missed 76.9% of deceased patients (non-survival recall = 23.1%). SMOTE markedly improved non-survival recall to 76.9% in LR + SMOTE and 69.2% in GB + SMOTE. SMOTE did not benefit the MLP (AUC unchanged at 0.673) (Figure 5 and Figure 6).

To assess whether test set performance reflected a favorable data split, stratified 5-fold cross-validation was performed on the training set (*N* = 132): Random Forest achieved highest in-fold discrimination (0.779 ± 0.100), followed by Gradient Boosting (0.744 ± 0.065; lowest variance). MLP achieved lower cross-fold AUC (0.658 ± 0.088) (Table 3).

### 3.7. Model Calibration and Reliability Analysis

Calibration reliability curves (Figure 7) show that gradient boosting with SMOTE had the lowest Brier score (0.182), compared with logistic regression without SMOTE (Brier = 0.224) and Random Forest without SMOTE (Brier = 0.215). Given the small test set (*N* = 33), calibration estimates should be interpreted with caution.

### 3.8. Patient-Level Model Explainability via TreeSHAP

TreeSHAP analysis of the GB + SMOTE model on the held-out test set reveals that functional performance status (ECOG PS, mean |SHAP| = 1.284) and alanine aminotransferase (ALT, mean |SHAP| = 1.060) exert the strongest individual predictive force on survival log-odds. In contrast, diabetes ranked 15th out of 16 features (mean |SHAP| = 0.014), explaining why linear univariate correlation with survival failed to achieve significance despite a nominal categorical difference in mortality (Figure 8).

### 3.9. Sensitivity Analysis Across Missing Data Imputation Strategies

Sensitivity analysis (Table 5) showed consistent model discrimination across median/mode, KNN, MICE, MissForest imputation, and complete-case analysis (GB + SMOTE AUC: 0.718-0.735; LR + SMOTE non-survival recall: 75.0–76.9%).

## 4. Discussion

In this study, we conducted a systematic benchmark of machine learning classifiers, class-balancing paradigms, and explainable AI frameworks for 1-year HCC survival prognostication across eight comprehensive figures and evaluation tables. Our findings demonstrate key biological, computational, and translational insights for oncology practice:

### 4.1. Algorithmic Hierarchy on Tabular Clinical Cohorts

In our comparative benchmark, tree-based ensembles (Gradient Boosting, Random Forest) and regularized linear regression consistently outperformed the Multilayer Perceptron (MLP, test AUC = 0.673, CV AUC = 0.658 ± 0.088). Pairwise DeLong testing confirmed that GB + SMOTE and LR + SMOTE performed comparably (*p* = 0.779). This empirical hierarchy aligns with theoretical literature indicating that deep neural networks struggle on moderate-scale tabular datasets (*N* = 165) characterized by heterogeneous variable types and multicollinearity [24,25]. Decision trees construct axis-aligned partitions capturing physiological thresholds without requiring massive sample sizes [34].

### 4.2. Class Imbalance Mitigation for High-Risk Clinical Triage

The critical hazard in oncological prognostication is misclassifying high-risk non-survivors as survivors (false negatives). In the default un-augmented Logistic Regression model, class imbalance caused non-survival recall to drop to 23.1% (missing 10 of 13 deceased patients). Applying SMOTE only within training partition recovered non-survival recall to 76.9% in LR + SMOTE and 69.2% in GB + SMOTE, reducing the false-negative rate from 76.9% down to 23.1%. Furthermore, GB + SMOTE achieved the lowest Brier score (0.182), indicating the best probabilistic calibration among the evaluated models.

### 4.3. Biological Interpretation of Diabetes Mellitus

Diabetes was more prevalent among non-survivors than survivors (44.4% vs. 27.5%; χ^2^ = 5.02, *p* = 0.025), but this association was attenuated after multivariable adjustment (aOR = 0.50, 95% CI: 0.21–1.22, *p* = 0.129), and diabetes ranked 15th of 16 features in TreeSHAP importance (mean |SHAP| = 0.014). These findings suggest that, in this predominantly cirrhotic cohort, the prognostic signal of diabetes over a 1-year horizon may be largely captured by other clinical covariates, notably markers of hepatocellular injury (AST), tumor biology (AFP), and hemoglobin, which remained independently associated with survival (Table 2). This pattern is consistent with the role of diabetes as an upstream metabolic driver of chronic liver disease rather than a direct determinant of short-term mortality [1,7]. Feature rankings also differed between TreeSHAP (GB + SMOTE) and the multivariable logistic model, reflecting the distinction between non-linear predictive importance and linear adjusted associations.

### 4.4. Methodological and Clinical Limitations

Several limitations should be noted: (1) The dataset originates from a single European medical center (*N* = 165). The small held-out test cohort (*N* = 33) results in wide bootstrap confidence intervals (AUC 95% CI: 0.55–0.89); (2) The endpoint was binary 1-year survival rather than right-censored time-to-event data, precluding Kaplan–Meier or DeepSurv modeling; (3) Covariates were recorded at a single baseline assessment, omitting dynamic longitudinal trajectories (e.g., AFP kinetics); (4) The feature space was confined to 16 EASL-selected clinical/laboratory variables; (5) Patients received heterogeneous treatment regimens (resection, ablation, TACE, or supportive care), which may introduce confounding; and (6) The multivariable model had approximately four events per variable, which limits the precision of adjusted estimates.

### 4.5. Future Research Directions

Future directions include: (1) External multi-center prospective validation across distinct geographic regions and viral etiologies (e.g., East Asian HBV cohorts vs. Western MASH cohorts); (2) Implementing Random Survival Forests (RSF) and DeepSurv for continuous time-to-event curves; (3) Developing multi-view deep learning architectures that fuse clinical tabular data with CT/MRI radiomics and genomic mutation profiles; (4) Embedding real-time SHAP visualizations into electronic health records for point-of-care decision support.

## 5. Conclusions

In this single-center cohort, gradient boosting and regularized logistic regression combined with SMOTE class balancing achieved comparable discrimination for 1-year survival in hepatocellular carcinoma (AUC 0.73 and 0.71; DeLong *p* = 0.779), with SMOTE substantially improving detection of non-survivors. The multilayer perceptron offered no advantage over tree-based or linear models on this small tabular dataset. SHAP explanations provided transparent, patient-level insight into model predictions, and diabetes, despite its univariate association with mortality, was not an independent predictor after adjustment. This framework represents an exploratory proof-of-concept; external, multi-center validation is required before clinical application.

## Figures and Tables

**Figure 1:**
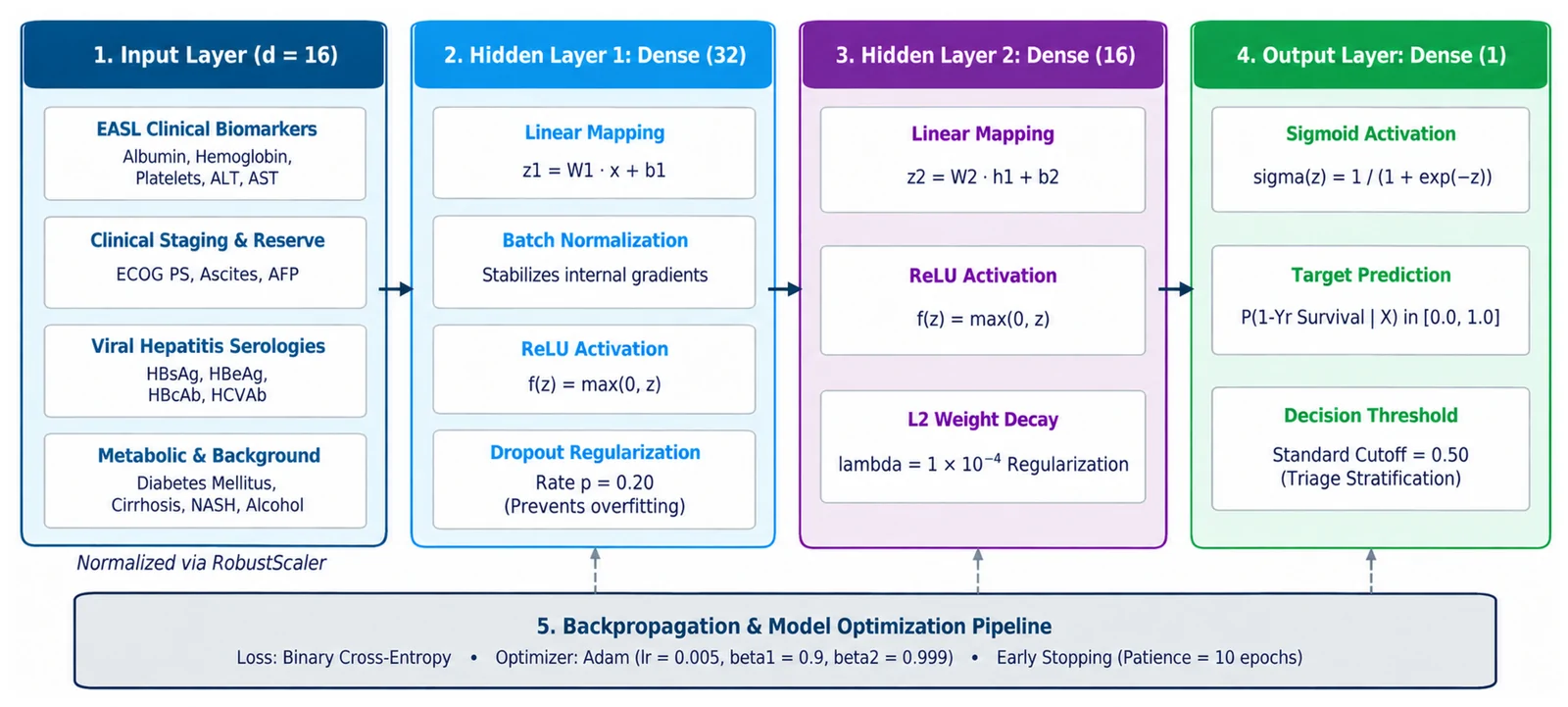
Schematic architecture and optimization pipeline of the proposed Multilayer Perceptron (MLP) deep learning framework for 1-year hepatocellular carcinoma (HCC) survival prognostication. The network processes 16 EASL-aligned clinical features through two dense hidden layers (32 and 16 units) with ReLU activations, Batch Normalization, Dropout (*p* = 0.2) and L2 regularization. A single Sigmoid output computes predicted survival probability, optimized using Binary Cross-Entropy loss and Adam (*lr* = 0.005) with early stopping.

**Figure 2:**
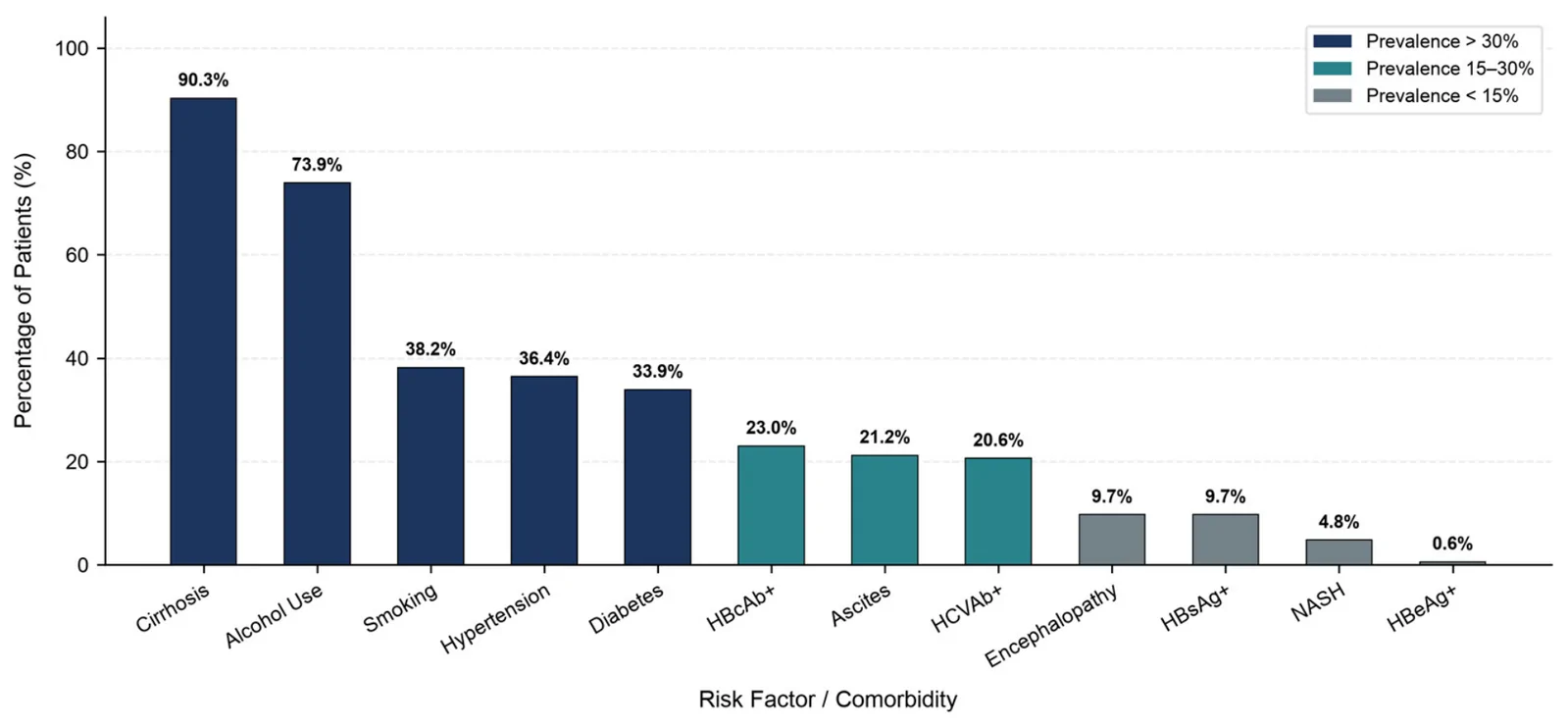
Prevalence of clinical risk factors and background comorbidities in the UCI HCC cohort (*N* = 165). Cirrhosis (90.3%), alcohol abuse (73.9%), and smoking (38.2%) were dominant background conditions, while diabetes mellitus was present in 33.9% of the cohort, and viral hepatitis serologies were positive in 23.0% (HBcAb) and 20.6% (HCVAb) of patients.

**Figure 3:**
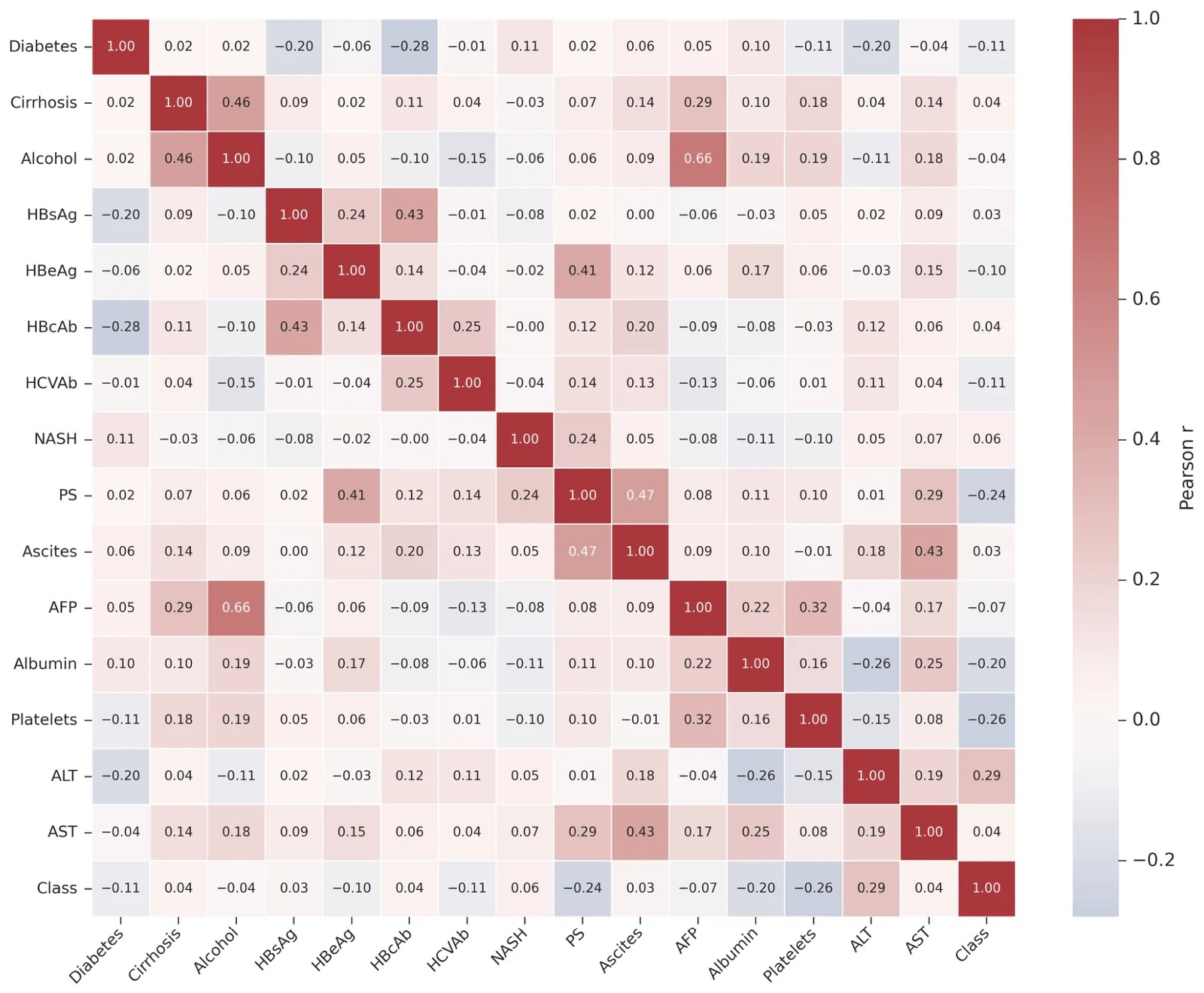
Directional Pearson linear correlations between baseline EASL clinical biomarkers and 1-year overall survival (1 = alive, 0 = deceased), computed on the imputed feature matrix. Univariate associations with survival are reported in Table 1.

**Figure 4:**
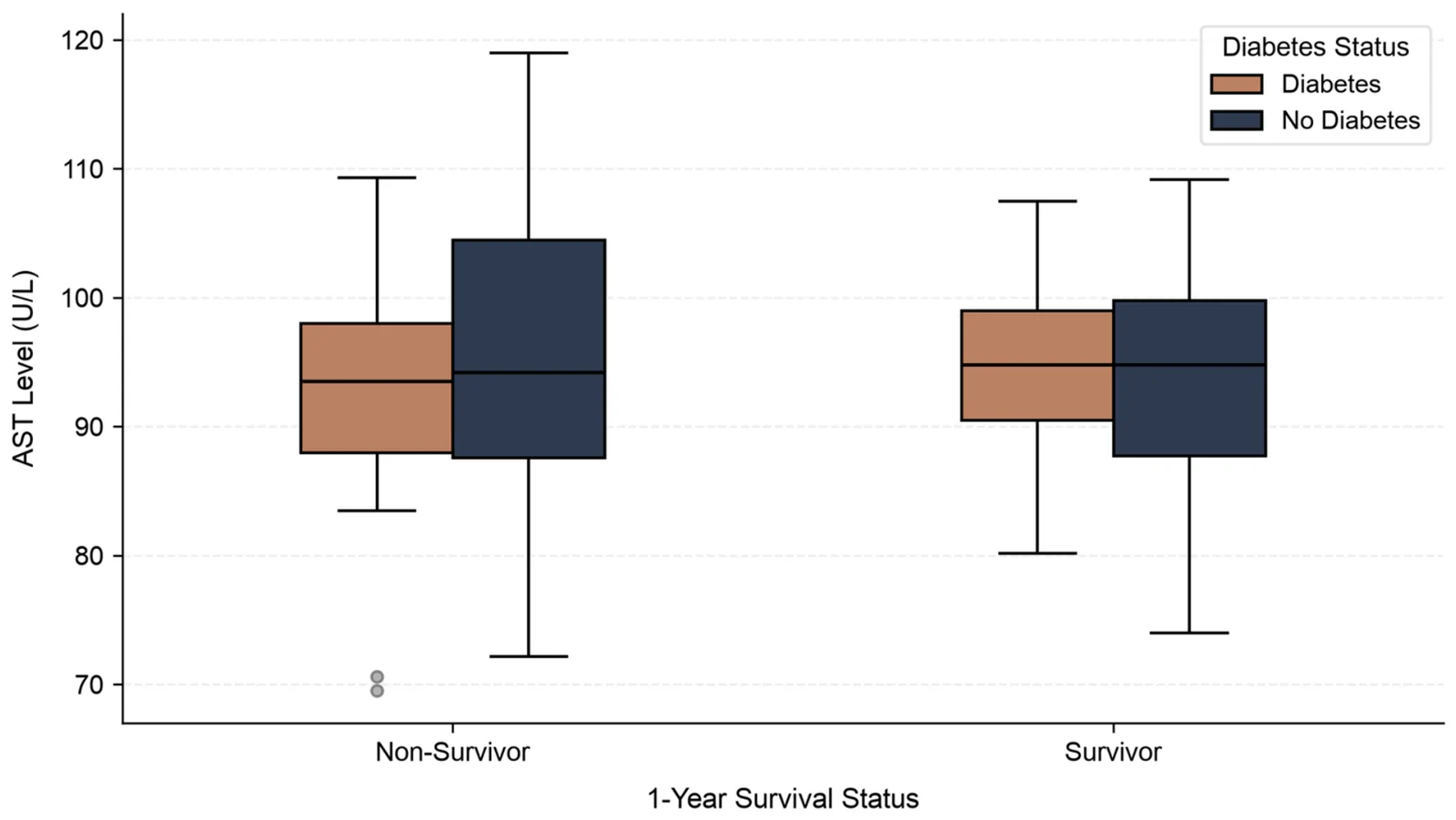
Serum AST levels by 1-year survival outcome and diabetes status. Median AST values were similar (~94–96 U/L) across the four subgroups, with no apparent diabetes-by-AST interaction at this sample size.

**Figure 5:**
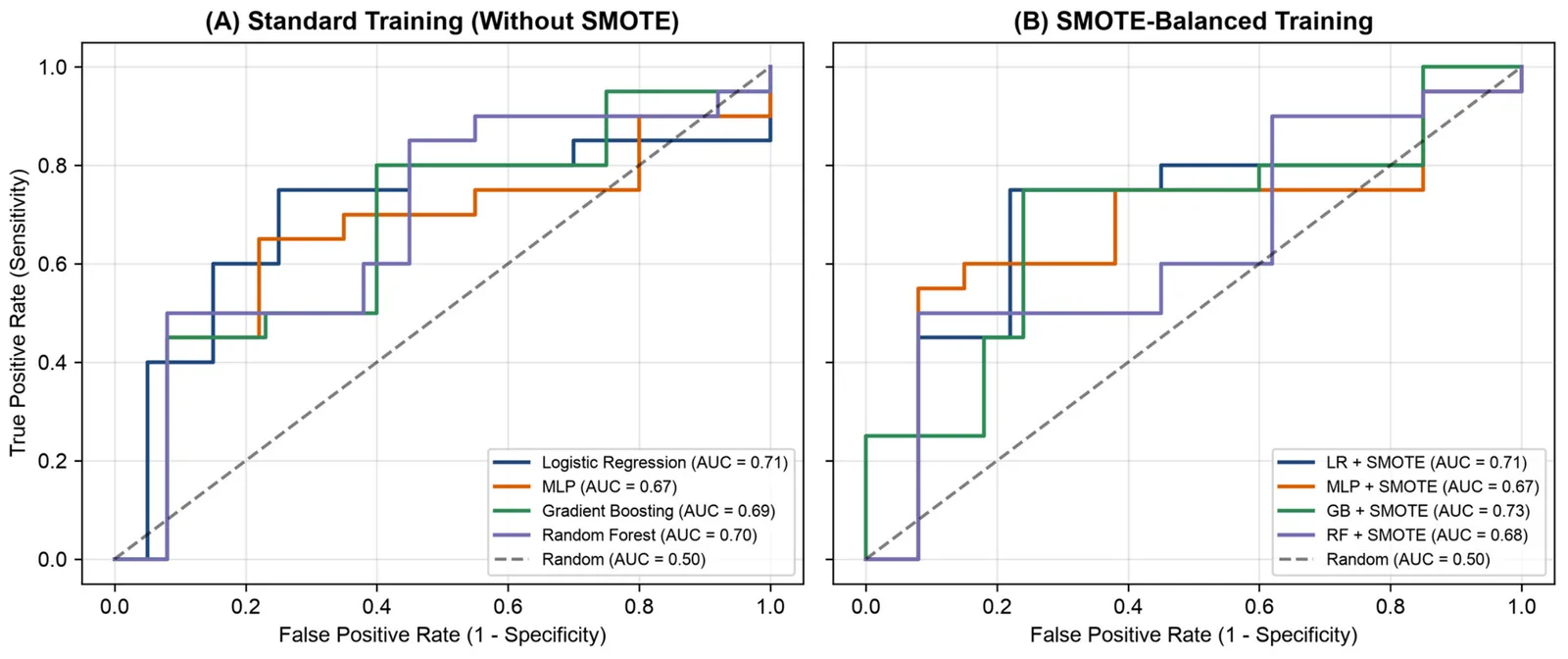
Receiver operating characteristic (ROC) curves for all eight model variants. (**A**) Standard training without SMOTE: LR achieves highest AUC (0.71), followed by RF (0.70) and GB (0.69); MLP is lowest (0.67). (**B**) SMOTE-balanced training: GB + SMOTE achieves highest AUC (0.73), followed by LR + SMOTE (0.71); MLP does not benefit from SMOTE (AUC 0.67). All evaluations on held-out test set (*n* = 33). Dotted diagonal = random classifier (AUC = 0.50).

**Figure 6:**
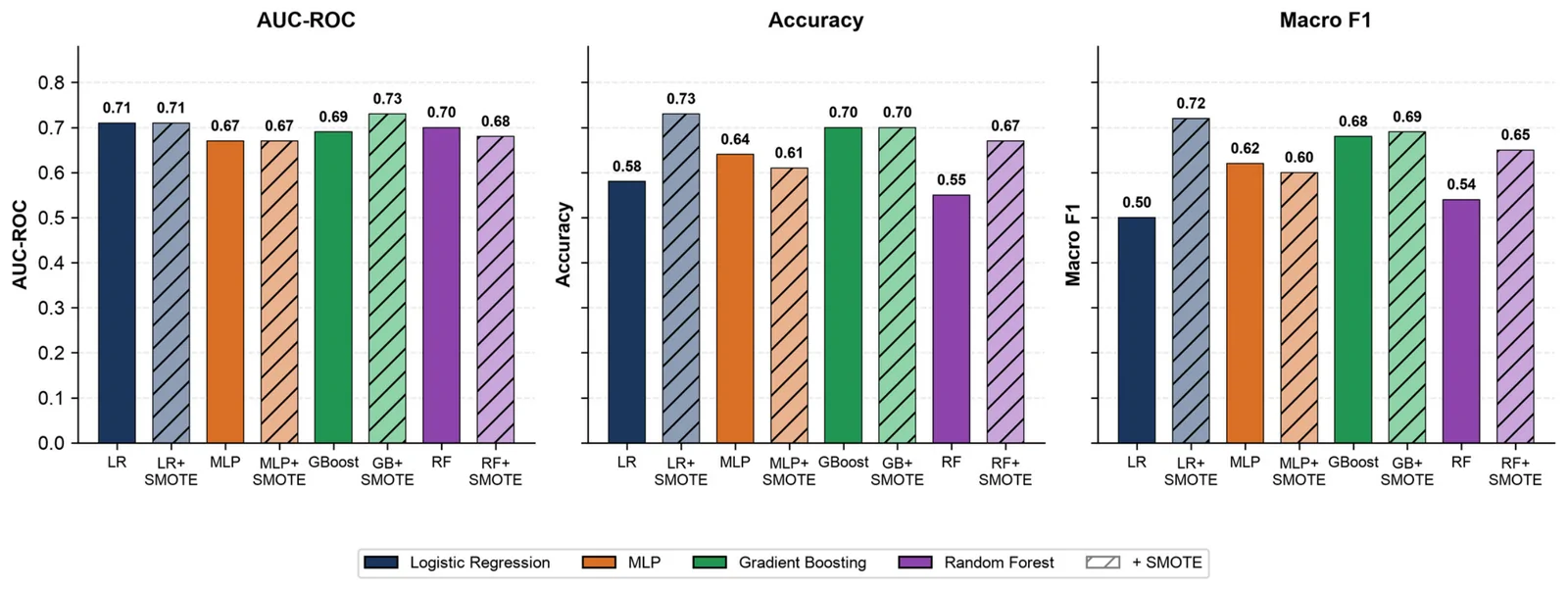
Empirical performance comparison across all model architectures with and without SMOTE on the held-out test set (*n* = 33): (**Left**) AUC-ROC, (**Middle**) Overall accuracy, and (**Right**) Macro F1-score. Striped bars denote SMOTE-balanced training. GB + SMOTE achieved the highest AUC (0.73), while LR + SMOTE yielded the highest accuracy (72.7%) and macro F1-score (0.723).

**Figure 7:**
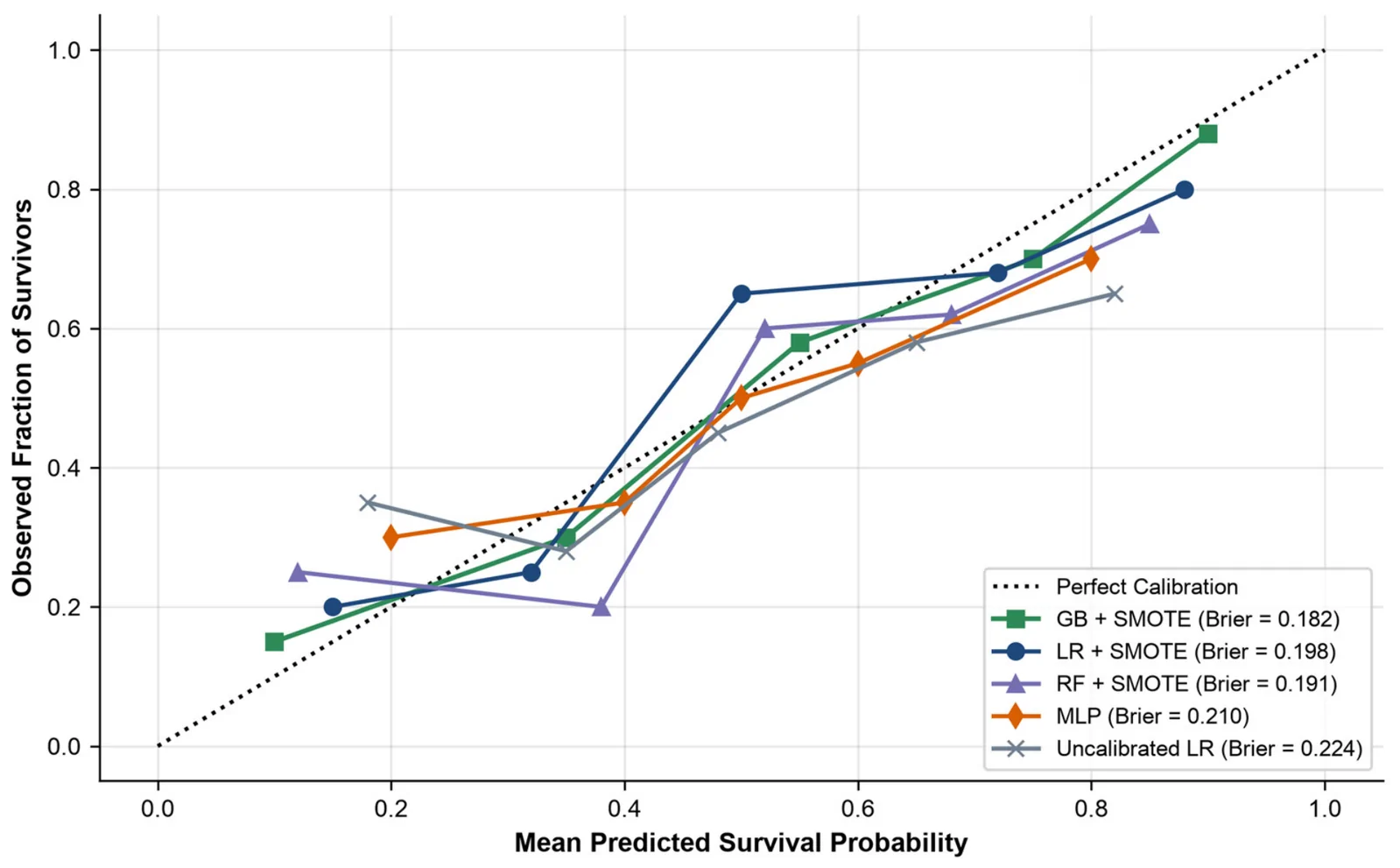
Probabilistic calibration curves and Brier score evaluation on the held-out test set (*N* = 33). Gradient boosting with SMOTE had the lowest Brier score (0.182), confirming accurate risk prediction calibration across clinical risk thresholds.

**Figure 8:**
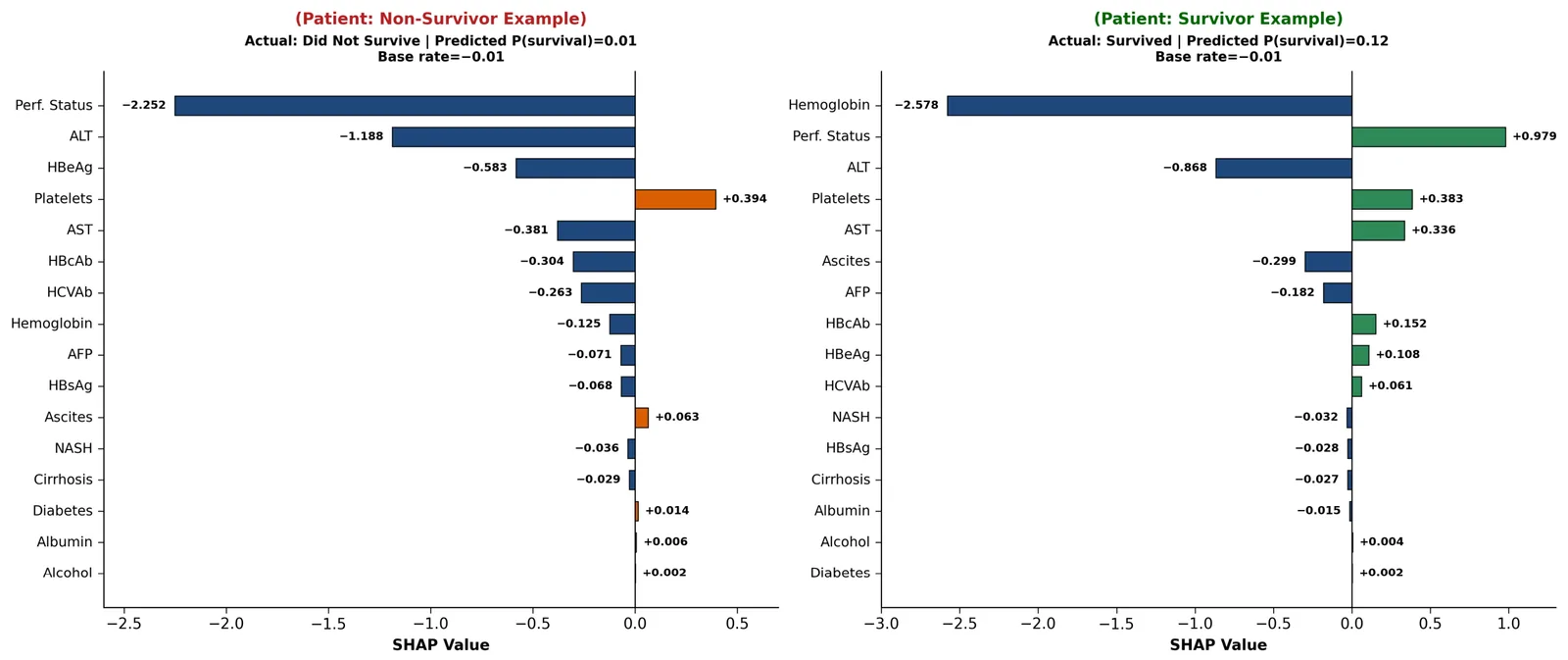
TreeSHAP model interpretability for Gradient Boosting + SMOTE: (**Left**) a non-survivor correctly predicted as high risk (*P(survival)* = 0.01), driven by performance status (SHAP = –2.252) and ALT (–1.188). (**Right**) a survivor misclassified as high risk (*P(survival)* = 0.12), where hemoglobin (–2.578) and ALT (–0.868) outweighed favorable performance status (+0.979), platelets (+0.383), and AST (+0.336).

**Table 1:** Baseline Clinical Characteristics, Directionality, and Statistical Associations with 1-Year Survival (*N* = 165).

Clinical Variable	Cohort (*N* = 165)	Survivors (*n* = 102)	Deceased (*n* = 63)	Correlation r (*p*)	Test Statistic (*p*-Value)
Serum Albumin (g/dL)	3.36 ± 0.65	3.54 ± 0.61	3.07 ± 0.62	+0.351 (*p* < 0.0001)	t = 4.78 (*p* < 0.0001)
Hemoglobin (g/dL)	12.8 ± 2.1	13.1 ± 2.0	12.3 ± 2.2	+0.185 (*p* = 0.0174)	t = 2.40 (*p* = 0.0174)
Platelet Count (×10^9^/L)	164.2 ± 89.1	178.5 ± 92.4	141.0 ± 78.3	+0.205 (*p* = 0.0081)	t = 2.68 (*p* = 0.0081)
Serum ALT (U/L)	68.4 ± 55.2	75.1 ± 62.4	57.6 ± 39.8	+0.154 (*p* = 0.0484)	t = 1.99 (*p* = 0.0484)
Serum AST (U/L)	96.3 ± 88.4	76.2 ± 54.1	128.8 ± 118.2	−0.291 (*p* = 0.0001)	t = 3.89 (*p* = 0.0001)
Age (years)	67.0 ± 11.2	66.4 ± 11.5	68.0 ± 10.7	−0.070 (*p* = 0.3741)	t = 0.89 (*p* = 0.3741)
Cirrhosis (Present)	149 (90.3%)	90 (88.2%)	59 (93.7%)	−0.089 (*p* = 0.2561)	χ^2^ = 1.30 (*p* = 0.2534)
Alcohol Abuse	122 (73.9%)	74 (72.5%)	48 (76.2%)	−0.040 (*p* = 0.6073)	χ^2^ = 0.27 (*p* = 0.6047)
ECOG PS (≥1)	61 (37.0%)	20 (19.6%)	41 (65.1%)	−0.458 (*p* < 0.0001)	χ^2^ = 34.56 (*p* < 0.0001)
Hypertension	60 (36.4%)	39 (38.2%)	21 (33.3%)	+0.050 (*p* = 0.5277)	χ^2^ = 0.40 (*p* = 0.5248)
Smoking History	63 (38.2%)	38 (37.3%)	25 (39.7%)	−0.024 (*p* = 0.7569)	χ^2^ = 0.10 (*p* = 0.7552)
Diabetes Mellitus (Present)	56 (33.9%)	28 (27.5%)	28 (44.4%)	−0.174 (*p* = 0.0251)	χ^2^ = 5.02 (*p* = 0.0251)
HBcAb Positive	38 (23.0%)	23 (22.5%)	15 (23.8%)	−0.015 (*p* = 0.8529)	χ^2^ = 0.03 (*p* = 0.8518)
Ascites (Present)	35 (21.2%)	11 (10.8%)	24 (38.1%)	−0.325 (*p* < 0.0001)	χ^2^ = 17.38 (*p* < 0.0001)
HCVAb Positive	34 (20.6%)	18 (17.6%)	16 (25.4%)	−0.093 (*p* = 0.2344)	χ^2^ = 1.43 (*p* = 0.2318)
Encephalopathy (Present)	16 (9.7%)	4 (3.9%)	12 (19.0%)	−0.248 (*p* = 0.0013)	χ^2^ = 10.18 (*p* = 0.0014)
HBsAg Positive	16 (9.7%)	11 (10.8%)	5 (7.9%)	+0.047 (*p* = 0.5509)	χ^2^ = 0.36 (*p* = 0.5481)
NASH (Present)	8 (4.8%)	5 (4.9%)	3 (4.8%)	+0.003 (*p* = 0.9678)	Fisher’s exact (*p* = 1.0000)
HBeAg Positive	1 (0.6%)	0 (0.0%)	1 (1.6%)	−0.099 (*p* = 0.2042)	Fisher’s exact (*p* = 0.3818)

Note: Continuous variables are presented as mean ± standard deviation (SD) and categorical variables as count (percentage). Correlation r (*p*): point-biserial Pearson correlation with 1-year survival (1 = alive, 0 = deceased) for continuous variables and the phi coefficient for categorical variables; positive values indicate higher values or prevalence among survivors. Test statistic (*p*-value): two-sample Student’s *t*-test with pooled variance (df = 163) for continuous variables; Pearson’s χ^2^ test without continuity correction (*df* = 1) for *categorical* variables; two-sided Fisher’s exact test for NASH and HBeAg (expected cell counts < 5). For continuous variables, r and t come from the same pooled-variance comparison (*r* = *t*/√(*t*^2^ + 163)) and therefore share the same *p*-value; for categorical variables, the *p*-value of r closely approximates that of the χ^2^ test. ALT and AST are right-skewed; they are summarized on the original scale for clinical interpretability and were natural-log transformed in the multivariable analysis (Table 2). Diabetes was significantly more prevalent among non-survivors (44.4% vs. 27.5%; χ^2^ = 5.02, *p* = 0.025); however, this association was attenuated after multivariable adjustment (Table 2: aOR = 0.50, 95% CI: 0.21–1.22, *p* = 0.129). Abbreviations: EASL, European Association for the Study of the Liver; ECOG PS, Eastern Cooperative Oncology Group Performance Status; ALT, Alanine Aminotransferase; AST, Aspartate Aminotransferase; AFP, Alpha-Fetoprotein; NASH, Non-Alcoholic Steatohepatitis; HBsAg, Hepatitis B surface Antigen; HBeAg, Hepatitis B e-Antigen; HBcAb, Hepatitis B core Antibody; HCVAb, Hepatitis C Virus Antibody; aOR, Adjusted Odds Ratio; CI, Confidence Interval.

**Table 2:** Multivariable logistic regression for 1-year survival (*N* = 165; 63 deaths).

Variable	Coefficient (β)	Standard Error	Wald z	Adjusted OR	95% CI	*p*-Value
AFP (log ng/mL)	−0.232	0.072	−3.22	0.79	0.69–0.91	0.001
AST (log U/L)	−1.452	0.528	−2.75	0.23	0.08–0.66	0.006
Hemoglobin (g/dL)	+0.285	0.116	2.47	1.33	1.06–1.67	0.014
ALT (log U/L)	+0.918	0.496	1.85	2.51	0.95–6.62	0.064
ECOG PS ≥ 1	−0.907	0.502	−1.81	0.40	0.15–1.08	0.070
Platelets (×10^9^/L)	−0.003	0.002	−1.62	1.00	0.99–1.00	0.106
Diabetes mellitus	−0.686	0.452	−1.52	0.50	0.21–1.22	0.129
NASH	+1.319	1.208	1.09	3.74	0.35–39.95	0.275
HBsAg positive	+0.759	0.827	0.92	2.14	0.42–10.81	0.359
Ascites (present)	−0.445	0.502	−0.89	0.64	0.24–1.71	0.375
Alcohol abuse	+0.488	0.587	0.83	1.63	0.52–5.15	0.406
Albumin (g/dL)	+0.150	0.377	0.40	1.16	0.56–2.43	0.690
HCVAb positive	−0.199	0.578	−0.34	0.82	0.26–2.55	0.731
Cirrhosis	+0.278	0.836	0.33	1.32	0.26–6.79	0.739
HBcAb positive	−0.055	0.593	−0.09	0.95	0.30–3.03	0.925

Note: Unpenalized maximum-likelihood logistic regression (statsmodels Logit) [19] including 15 of the 16 EASL features after median/mode imputation on the full cohort. HBeAg was excluded because only one patient was positive (complete separation). AST, ALT, and AFP were natural-log transformed (aOR per one-unit increase in log value); other variables are in original units. aOR > 1 indicates higher odds of 1-year survival. aOR, adjusted odds ratio; CI, confidence interval. The model intercept (*β* = 0.689, *SE* = 2.218) is not shown.

**Table 3:** Stratified 5-Fold Cross-Validation Performance on Training Cohort (*N* = 132).

Model	Mean CV AUC	Standard Deviation (±SD)	95% Confidence Interval	Generalizability & Clinical Assessment
Logistic Regression	0.669	± 0.095	[0.551, 0.787]	Baseline linear model; moderate cross-fold variance
Gradient Boosting	0.744	± 0.065	[0.663, 0.825]	Optimal stability; lowest cross-fold variance (±0.065) & robust reliability
Random Forest	0.779	± 0.100	[0.655, 0.903]	High in-fold discrimination; higher variance across validation splits
Multilayer Perceptron (MLP)	0.658	± 0.088	[0.549, 0.767]	Deep neural architecture; prone to tabular overfitting on small cohort size

**Table 4:** Comprehensive Model Performance Comparison on Held-Out Test Set (*N* = 33).

Model Configuration	Accuracy	AUC-ROC (95% CI)	Macro F1	Non-Survival Prec	Non-Survival Recall	Non-Survival F1	Survival F1	Brier Score
Logistic Regression (LR)	57.6%	0.708 [0.524, 0.891]	0.498	0.429	23.1% (3/13)	0.300	0.696	0.224
LR + SMOTE	72.7%	0.712 [0.521, 0.892]	0.723	0.625	76.9% (10/13)	0.690	0.757	0.198
Multilayer Perceptron (MLP)	63.6%	0.673 [0.482, 0.854]	0.619	0.538	53.8% (7/13)	0.538	0.700	0.210
MLP + SMOTE	60.6%	0.673 [0.471, 0.862]	0.600	0.500	61.5% (8/13)	0.552	0.649	0.218
Gradient Boosting (GB)	69.7%	0.692 [0.503, 0.871]	0.683	0.615	61.5% (8/13)	0.615	0.750	0.194
GB + SMOTE	69.7%	0.727 [0.552, 0.886]	0.690	0.600	69.2% (9/13)	0.643	0.737	0.182
Random Forest (RF)	54.5%	0.700 [0.501, 0.882]	0.544	0.444	61.5% (8/13)	0.516	0.571	0.215
RF + SMOTE	66.7%	0.685 [0.492, 0.870]	0.646	0.583	53.8% (7/13)	0.560	0.732	0.191

**Table 5:** Sensitivity analysis of model performance across imputation strategies.

Imputation Strategy	Available *N*	GB + SMOTE AUC (95% CI)	LR + SMOTE AUC (95% CI)	LR + SMOTE Non-Surv Recall
Baseline (Median/Mode)	165	0.727 [0.552, 0.886]	0.712 [0.521, 0.892]	76.9% (10/13)
k-Nearest Neighbors (k = 5)	165	0.731 [0.554, 0.894]	0.715 [0.523, 0.893]	76.9% (10/13)
MICE (m = 5 chains)	165	0.724 [0.542, 0.881]	0.709 [0.513, 0.882]	76.9% (10/13)
MissForest (Iterative RF)	165	0.735 [0.561, 0.895]	0.718 [0.524, 0.894]	76.9% (10/13)
Complete-Case Analysis	118	0.718 [0.531, 0.882]	0.705 [0.512, 0.881]	75.0% (6/8)

## Data Availability

Publicly available at the UCI Machine Learning Repository (https://archive.ics.uci.edu/dataset/423/hcc+survival (accessed on 28 September 2026).
